# Protonuclein in General Practice1Read before the Detroit Medical and Library Association.

**Published:** 1899-01

**Authors:** G. W. Sherman

**Affiliations:** Detroit, Michigan


### PROTONUCLEIN IN GENERAL PRACTICE.[1]

By G. W. SHERMAN, M. D., Detroit, Michigan.

(*From The Physician and Surgeon.*)

. . . . . . . . .

MY FIRST practical experience with protonuclein was on myself. About two and a half years ago I was taken with a severe attack of acute catarrhal inflammation of the nasal mucous

---

1. Read before the Detroit Medical and Library Association.

membrane, which rapidly extended down the trachea into the bronchi. It began on Friday morning with an almost incessant sneezing, accompanied by blocking of the nose, fullness in the head and headache, followed later in the day by a thin, copious discharge from the nose, and an irritating cough. By 5 o'clock p. m. the same day my headache was severe, my limbs all ached, and on taking my temperature it registered 101°. I had had similar attacks before, none apparently quite so severe, which always run a course of from one to three weeks. I had tried quinine and other remedies without any appreciable benefit, and was a willing subject to try something new. I had a few samples of protonuclein and began to take them *ad libitum*, starting about 5 o'clock in the evening. By Saturday morning I felt some better and continued taking the preparation through all that day, still *ad libitum* and by evening, twenty-four hours after I began its use, felt considerably improved. I continued taking more during Sunday, when my nose cleared up, and the headache, fever, cough and soreness in my limbs disappeared. By Monday evening, after three days' treatment, I was practically well and attended a meeting of the Detroit Medical and Literary Association. Since then I have always prescribed protonuclein in these acute catarrhal affections with the same happy result. Experience has taught me that the proper dose for such cases, in the adult, is from six to twelve grains, repeated every two to three hours. The treatment should be continued with smaller doses for a few days after the disease has disappeared to prevent a relapse.

I have found protonuclein especially useful in the treatment of broncho-pneumonia in infants and children. In these cases I generally give from two to four grains, according to age, repeated every two to three hours, and find that a recovery takes place in from three to five days. I have had remarkable success in treating pneumonia with this preparation and will briefly report three cases:

CASE I.—My mother, aged 72 years, on April 8, 1897, suffered a severe chill about 9 o'clock in the evening. Two hours later, when I first saw her, she complained of pain in the right side, was coughing up bloody mucus, and was very uneasy. Her heart had been irregular for some years, but now the pulse was 130 and her temperature 103°. Physical examination revealed pneumonia of the right lung. I prescribed two grains of phenacetin and six grains of protonuclein, to be repeated every two hours. By 10 o'clock the next day her temperature was 99 3-5°, and her pulse 108; the pain in her side was less, and she felt much better. The phenacetin was discontinued and the protonuclein continued. By the third day her temperature was normal, and she felt so well that, in spite of my protests, she was determined to sit up. She coughed up rust-colored sputum for six or seven days, but otherwise felt quite well. She has had no trouble with her lungs since.

CASE II.—C. G., a male, aged sixty-three years, had not felt well for several days, and was taken with a fever the day before I saw him. Patient complained of pain in his right side and difficulty in breathing. His temperature was 102 3-5°, pulse 110, and the lower portion of his left lung was inflamed. I prescribed six grains of protonuclein, and ordered that the dose be repeated every two hours. The next day there was hepatization of the lower half of the right lung, with a temperature of 102° and a pulse of 108. The protonuclein was now increased to nine grains, repeated every two hours. The third day the temperature was 101° and the pulse 100. He felt better, and on examination the lung was found to be clearing up. The protonuclein was continued. On the fourth day the temperature was 98°, the pulse 84, patient had enjoyed a night's rest, appetite returning, and lung much improved. The fifth day I found my patient dressed and sitting in a chair. He said he felt well, but I persuaded him to go back to bed again, fearing something might happen. I continued the protonuclein four times a day for a few days, when he made a complete recovery.

I have treated ten cases of typhoid fever with protonuclein, all of which made an unusually early recovery, considering the severity of the early symptoms of some cases.

. . . . . . . .

I was called to a family in which one of the city physicians had charge of two typhoid fever cases; one, aged twenty years, who had been sick three weeks, and another, aged six years, who was just convalescing after seven weeks' illness. By the time I made my second call, a few days later, two other children of the family had taken sick. A boy seven years of age had not been feeling well for a few days, had no appetite, felt tired, tongue dry and coated, temperature 101°. I gave him four grains of protonuclein every three hours. He began to feel better in a few days, and by the eighth day had entirely recovered. I will leave the members to decide whether this was typhoid fever or not. The other case was a girl aged 10 years. She had the usual symptoms of typhoid fever, with a temperature of 102½°. Protonuclein, six grains, and phenacetin, two grains, repeated every three hours, were prescribed. The temperature continued to rise until the fifth day, when it reached 104 1-5°, pulse 130. The phenacetin was discontinued and the cold pack substituted (which was poorly dispensed), and protonuclein increased to nine grains, repeated every two hours. The temperature from the fifth to the tenth day ranged between 102½° and 104½°, and considerable diarrhea set in, which was controlled with bismuth and turpentine emulsion. From the tenth day the temperature gradually declined until the fifteenth day, when it became normal and remained so thereafter. It will be noticed that larger doses of protonuclein were used in this case than in the first case, and a more decisive recovery ensued.

I have recently treated two other patients, one aged six years and the other twelve years, both girls, with large doses of protonuclein, in whom the fever run a course almost identical with the above case. The one unusual feature in these three cases was the early appearance of the appetite. About the twelfth or thirteenth day they began to ask for food, and in a few days the desire to take nourishment became so keen that it was difficult to refuse them something more substantial than milk. All these cases lost their hair during convalescence.

Protonuclein has a wonderful effect in maintaining the spirits and vitality of a patient during fever and has no depressing effect, while it reduces the temperature. This is particularly noticeable in typhoid cases. They do not lapse into that stupid condition which is so characteristic of this disease.

When protonuclein is taken in large doses, say ten to fifteen grains repeated every two or three hours, it produces a deafness and ringing in the ears very similar to that produced by large doses of quinine. In such doses it may also cause an unsteadiness of the nerves and an increased frequency of the heart's action. If this condition is observed during the treatment of a disease it is well to withhold a few doses, when these symptoms will readily disappear without leaving any bad effects.

I have given protonuclein in scarlet fever with the effect of having the temperature decline and the swelling of the glands of the neck disappear while the rash is coming out. I have given it with great success in puerperal fever, erysipelas, infected wounds, and in fact consider it a valuable remedy in all infectious diseases.

Protonuclein also has quite marked tonic effects which are particularly noticeable when given in cases of general debility resulting from advanced age. As a tonic it should be given in from six to nine grain doses after meals and at bedtime. In neurasthenic cases it is of benefit, restoring a normal tone to the nervous system. I have given it in a few cases of whooping-cough with benefit. I have given it to a few tubercular cases, but cannot say that it was followed by especial improvement. In cases wherein the temperature is high I usually prescribe small doses of phenacetin as a palliative remedy to assist in bringing down the temperature until the protonuclein has time to produce results. I consider protonuclein a very valuable addition to our remedies in combating disease, and feel that all who use itin large doses will be gratified with the results.—*Physician and Surgeon.*

---

### TREATMENT OF CHOREA.

Dr. Moncorvo in a recent paper (*Pediatrics*) states that the frequency with which nervous and other symptoms are met with in chorea, places beyond doubt their influence as etiological factors. Hysteria also appears to exercise a marked influence on the onset of the disease, while it would seem possible that it is sometimes the sequel of the infectious disease common to childhood. A still closer connection appears to exist between chorea and rheumatism, so as to lead the author to see in chorea only the cerebro-medullary tendency of a rheumatic infection developed in a hysterical or neurasthenic temperament. Of the numerous remedies to which resort has been made in the treatment of chorea, he prefers those derived from the aromatic plants, and especially quinalgen, whose curative action in its treatment he was the first to demonstrate. Moncorvo reports in full the history of eight cases in which this drug was used with very successful results. It was administered in tablet form, in doses varying from a half to four grams in twenty-four hours, and was invariably well tolerated. Sometimes it was found necessary to increase the dose, but no inconvenience therefrom was ever experienced.

---

### CRAIG COLONY PRIZE FOR ORIGINAL RESEARCH IN EPILEPSY.

THE president of the board of managers of Craig Colony offers a prize of $100 for the best contribution to the pathology and treatment of epilepsy, originality being the main condition.

The prize is open to universal competition, but all manuscripts must be submitted in English. All papers will be passed upon by a committee to consist of three members of the New York Neurological Society, and the award will be made at the annual meeting of the board of managers of Craig Colony, October 10, 1899. Each essay must be accompanied by a sealed envelope containing the name and address of the author and bearing on the outside the motto or device which is inscribed upon the essay.

The successful essay becomes the property of the Craig Colony, for publication in its annual medical report.

Manuscripts should be sent to Dr. Frederick Peterson, 4 W. 50th Street, New York City, on or before September 1, 1899.—*Craig Colony Press.*

---

DR. ALBERT ABRAMS, author of the "Antiseptic Club," has written a series of satirical sketches which abound in rich humor and are pronounced his best work. They will appear in the *Medical-Fortnightly*, beginning January 1st, under the head of "Scattered Leaves from a Physician's Diary."

